# An Account of the Beneficial Effects of Ergot in a Case of Hydatids of the Uterus

**Published:** 1828-07

**Authors:** P. B. Johnson

**Affiliations:** M‘Connelsville, Ohio


Art. III.—*An account of the beneficial effects of* Ergot, *in a case of Hydatids of the Uterus, By* Dr. P. B. Johnson, *of M^c Connelsville, Ohio.*

In the first number of the American Journal of the Medical Sciences, there is an account of the use of ergot in a case of hydatids of the Uterus; communicated in a letter from Dr. J. W. Anderson to Dr. Dewees. By this letter it appears, that this is the first case of that disease, wherein the ergot is known to have been used. The following is a short statement of a case that happened in my own practice.

Mrs. W., aged twenty four years, of good constitution, was taken, about the 1st of September, 1825, with pain in her abdomen, attended with a discharge from the vagina, of a light coloured fluid, loss of appetite, some fever, and occasional nausea. For four months she had supposed herself pregnant, and was at this time nearly as large as a woman who had gone her full time. The physician who attended, believed her to be dropsical, and had prescribed accordingly. On the 15th of September, I was requested to visit her.—When I arrived, I found her extremely exhausted, with a pulse scarcely perceptible, and a copious flooding, which still continued. Upon examination, *per vaginam*, I discovered in the mouth of the uterus, something which I supposed to be clotted blood; but upon removing my hand, I found, sticking to the ends of my fingers, a number of small vesicles filled with a transparent lymph, which convinced me that her disease was hydatids of the uterus; and, inasmuch as the os uteri was not sufficiently dilated to permit me to introduce my hand, and her pains were but slight, I determined to try the effects of ergot, although I had never heard of its use in this disease. I gave her between 15 and 20 grains, and in 20 minutes her pains became regular and strong; the flooding abated *considerably*, and in a short time, she discharged, in detached pieces, nearly a gallon of hydatids, varying from the size of a mustard seed to that of a cherry. Her recovery was slow; and in January following, she was taken with a violent menorrhagia, which proved very obstinate; but at length, she regained her usual health, and has since been delivered of a perfect child. The above is the only case of hydatids of the uterus that I have seen; and I feel confident, that if the ergot had not been used, it would have proved fatal.

*M'Connelsville, Ohio, July*, 1828.

